# A Dynamic Motion Analysis of a Six-Wheel Ground Vehicle for Emergency Intervention Actions

**DOI:** 10.3390/s21051618

**Published:** 2021-02-25

**Authors:** Lucian Ștefăniță Grigore, Damian Gorgoteanu, Cristian Molder, Octavian Alexa, Ionica Oncioiu, Amado Ștefan, Daniel Constantin, Marin Lupoae, Răzvan-Ionuț Bălașa

**Affiliations:** 1 Military Technical Academy, “FERDINAND I”, 39–49 George Coșbuc Av., 050141 Bucharest, Romania; lucian.grigore@gmail.com (L.Ș.G.); damian.gorgoteanu@mta.ro (D.G.); cristian.molder@mta.ro (C.M.); alexa.octavian@gmail.com (O.A.); amado.stefan@mta.ro (A.Ș.); daniel.constantin@mta.ro (D.C.); marin.lupoae@mta.ro (M.L.); balasa.razvan@rocketmail.com (R.-I.B.); 2 Faculty of Finance-Banking, Accountancy and Business Administration, Titu Maiorescu University, 040051 Bucharest, Romania

**Keywords:** mobile robotics, sensors, mobility, wheel, kinematic, dynamic, rescue

## Abstract

To protect the personnel of the intervention units operating in high-risk areas, it is necessary to introduce (autonomous/semi-autonomous) robotic intervention systems. Previous studies have shown that robotic intervention systems should be as versatile as possible. Here, we focused on the idea of a robotic system composed of two vectors: a carrier vector and an operational vector. The proposed system particularly relates to the carrier vector. A simple analytical model was developed to enable the entire robotic assembly to be autonomous. To validate the analytical-numerical model regarding the kinematics and dynamics of the carrier vector, two of the following applications are presented: intervention for extinguishing a fire and performing measurements for monitoring gamma radiation in a public enclosure. The results show that the chosen carrier vector solution, i.e., the ground vehicle with six-wheel drive, satisfies the requirements related to the mobility of the robotic intervention system. In addition, the conclusions present the elements of the kinematics and dynamics of the robot.

## 1. Introduction

An alarming increase in risks has recently been observed, coming from both natural and artificial sources. Among the risks caused by technological hazards, the most frequent and significant risks are estimated for fires and radiation, depending on their frequency, complexity, and consequences [1,2]. The risks caused by natural causes, which contribute to the worsening of emergencies, include climate change as in the “Hyogo Framework for Action”. Response personnel in reported cases [3,4] are permanently at risk, which could even include death, regardless of the equipment and substances used to protect them from the effects of radiation and fire [5,6,7,8]. The multiple causes of death of firefighters during missions include smoke inhalation, burns, crush injuries, and trauma [9].

In previous studies, some authors have described the design shortcomings of pre-existing machines. The design errors of the intervention robots are mainly due to the lack of standards for their geometric aspects. The size of an intervention robot must take into account certain factors determined by the characteristics of the missions it must perform. As no standardization is available to facilitate the preparation of specifications, we focused our research on proposing an intervention robot system. The proposed system is an integrated model, with a propulsion/transport platform and an operational platform, which allows the simultaneous acquisition of data and their transmission to a ground control station (GCS) with movement in the area of action, which allows data analysis, decision making, and autonomous intervention. For the design of the robotic system, a terrestrial robotic system can be considered effective if: it can move autonomously in the area with maximum risk, can investigate and monitor the amount and concentration of dangerous substances or radiation, and can intervene in reducing/eliminating the source of risk [10,11,12]. The research work on the development of a family of specialized robots for emergencies is a continuation of the research carried out so far [13].

As we found no references in the studied literature to the use of specialized programs and methodologies for the evaluation of autonomous mobile robots in extreme environments with high temperatures, we used a simulation of a 6 × 6 vehicle model for which the experimental data were capable of validating the simulation methods adopted. The main requirements for experimental activities are a high degree of accuracy, a high degree of detail of the samples, repeatability of environmental conditions, and the ability to interface with dedicated programs for data analysis. These often-contradictory requirements become extremely complex in situations with high temperatures, fluctuations in wind direction, and the specificity of radioactive elements depending on the risk area.

In this context, the problem we aimed to address was: What is the optimal solution for developing an autonomous robotic system on wheels that can perform intervention/rescue missions in risky situations?

Motivated by this question, our aim was to analyze and validate an analytical-numerical model (2D kinematics and 3D dynamics) for the carrier vector. This carrier vector is a 6 × 6 wheeled vehicle. We developed an analytical-experimental model regarding the kinematics and dynamics of an Unmanned Ground Vehicle (UGV) with multiple uses for use in emergency situations. Each of the four theses will determine the status of the robot, corresponding to the stage of development.

To perform the analysis, the main directions of this study are summarized as follows:

- Limitations on the progression of the robot under the conditions imposed by missions to detect the level of radiation
- The level of radiation that may affect the communication system or the quality of the data captured by the sensors
- Identification of obstacles
- Reserve of energy resources

The task formulation for our research refers to the following:

- Imperfections due to the execution of the robot, which was constructed in the laboratory
- Discussing a robotic system (carrier vector and operational vector), which, to complete, requires four main stages:
  ○ Establishing the analytical-numerical model to achieve the kinematic and dynamism performance required to complete the missions presented above
  ○ Developing a navigation model that avoids obstacles
  ○ Improving operational platforms
  ○ Testing and evaluation of the robotic system

Regarding the novelty of the models, the integration of the elements of kinematics and vehicle dynamics with those related to the communications system and those due to emergencies (fires, radiation, etc.) is a subject that has not yet been integrated (in the literature). To the best of our knowledge, there are no autonomous robots that can fully perform intervention tasks in emergency situations while simultaneously performing those related to movement, orientation, bypassing obstacles, etc. As such, a hardware and software (H&S) solution for a future modular intervention robot (carrier vector and operational vector) will allow for a wide range of interventions at reasonable costs [14].

At this stage of project development, the integration will be done at a software level. The three types of controllers used are dependent on their own operating system. Also, the development of a family of robots with a transport platform and an operational one (depending on the missions) will represent the novelty of the robot. For now, companies producing robotic disaster response systems are making robots for one type of intervention. The solution proposed in the article would be able to participate in various missions only by changing the operational platform. In addition, each subsystem could be replaced on the principle of very flexible and agile assembly. The concept “Plug and Execute” allows the easy and quick insertion and removal of a new device. The concept was designed by analogy with the Plug and Play concept used in computers. The proposed concept could greatly increase the agility of emergency response systems.

The paper is structured as follows: Section 2 addresses the configuration of the intervention robot: hardware, controller, external sensor system, and propulsion system; Section 3 presents the algorithm for the analytical determination of the kinematics and dynamics (2D and 3D) characteristics. Section 4 provides the model for simulating the variation of the slope climbing, crossing the step type obstacle. The experimental methods used to determine the variation in the deceleration and acceleration while braking on a slope are presented in Section 5. Section 6 presents two of the risky emergency situations in which the robot can intervene: fires and gamma radiation emissions. Section 7 presents the conclusions and future developments.

## 2. Configuration of the Intervention Robot

### 2.1. Hardware

In this section, ‘Hardware’, we define all the components that would allow the robot to perform the actions required for different tasks [15], either commanded or autonomous [4,16].

**Figure 1:**
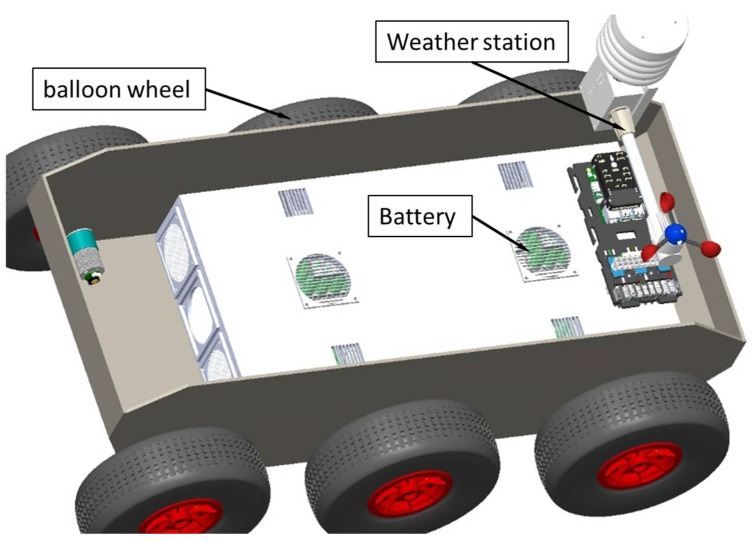
The terrestrial 6 × 6 robot developed for identifying, monitoring, and intervening in risky areas. The structural elements of the robot include the command-and-control system, the battery system, and the weather station.

Following the successive iterations of development (three so far), the hardware architecture of the robot in our research presented in Figure 1 allowed us to continue our research by developing the two vectors: carrier and operational. It is possible to mount various operational platforms on the housing cover: (1) for extinguishing fires; (2) to determine the level of radiation; and (3) a manipulator arm for carrying out interventions in case of artisanal explosive devices.

The literature suggests that a robot has artificial intelligence if the following hold:

- Its perception is constituted from a system of sensors, controllers, and software modules that are essential for moving from point A to point B. The perception system detects, classifies, and localizes natural and artificial features of the unstructured environment in which it moves. The specific objectives of the perception system for crossing a path, following a planned path and avoiding the obstacles, come from the necessary velocity of the vehicle and from the characteristics of the presumed working environment (the density of the obstacles, visibility, illumination, weather, and the edges of the road) so that the UGV can determine the corresponding velocity, stop, or avoid obstacles [4,17].
- When planning the paths that will be followed, a path is generated with no collisions in an environment with obstacles, and the path is optimized by respecting some criteria. This deals with the planning methods of global and local paths. The planning methods use the data captured by the sensors to estimate the parameters of the environment and moving the robot in real-time. The global method needs a completely known environment and certain simplifying methods; for the local method, the algorithm allows some real-time adjustments to the path to be followed. Planning implies optimization procedures of the time (and velocities) to select the geometrical paths in real-time to avoid obstacles [18,19,20,21]. Since every obstacle creates a risk level for the UGV, introducing proportional integrative derivative (PID) controllers and fuzzy logic methods, which classify the objects around the vehicle based on their level of risk, allows generating some predictions regarding the capacity to avoid fixed or moving obstacles (the velocity obstacle (VO) approach), which means that, virtually, a space that defines the respective object should be generated [22,23,24].
- The mission of a UGV agrees with path planning through adopting some algorithms that can generate optimal behavior and, each time discrepancies from the initial map appear, these data are updated. Thus, the error and cost functions increase the efficiency when crossing a certain space, and the search would become faster [25,26].
- The wireless communication and tele-command of a UGV with a ground control station (GCS) with an operator or with another robot are achieved through wireless mesh networks (WMNs). These networks are self-organized and dynamically auto-configured. For quality communication, these systems have scalability and security as objectives [27,28]. Tele-command is very important [28], even though its functionalities are due to its sensors; the video Infra-Red/Electro-Optical (IR/EO) devices and any other attached devices allow the sampling of data [29,30,31].
- The navigation system of a robot is a non-structured environment that uses path planning, obstacle avoidance and circumnavigating, localization, and perceptive interpretation techniques. All these cannot be combined in a single system (a single software application), which is why for each robotic system, a custom project must be realized with a software system that should be adapted to the respective application. A navigation architecture becomes efficient if the mobility of the robot is divided into specialized software modules. This architecture consists of software modularity (as a consequence of introducing a new sensor or maintaining some obstacle avoidance modules based on certain cinematics), robot location control based on the different functionalities and learning algorithms, techniques of time-domain analysis (response time of the sensors, temporal depth, space localization, and decision making based of the dynamics of the robot), and decoupled control [32,33,34,35,36].
- Implementing machine learning and deep learning techniques requires precise information so that the path planning allows locating the robot in space and memorizing the position of obstacles. UGVs are complex autonomous systems that use artificial intelligence (AI) through image recognition, human-machine interaction, intelligent decisions, logic, and learning. The deep learning algorithms restricted Boltzmann machines (RBMs), and convoluted neural networks (CNNs) are specially used for computer vision (CV) applications for object recognition. The data quantity necessitates using algorithms of the back-propagation types to compute the gradients and stochastic gradient descent optimization methods to minimize the errors in the outputs from the network as a function of the corresponding inputs. Apart from these, various auto-encoders, capsule networks, and synthetic gradients are needed, which we will discuss in future research in the domain of UGVs [37,38,39,40,41,42].
- Obstacles are defined as the elements that appear on the robot’s path—bumps and ditches are just elements of the road that are described in the equations of the contact of the engine with the ground.

The research part on autonomous movement (machine learning, deep learning, etc.) is already underway. The reviewers’ suggestions in this regard will be considered. For route planning, we think it is much better to focus on route planning algorithms such as Rapidly-Exploring Random Tree (RRT), Lattice, and the A* search algorithm.

### 2.2. The Controller

The implemented controllers integrate the systems, and the data flow was commercial off-the-shelf (COTS) accessible [43,44].

**Figure 2:**
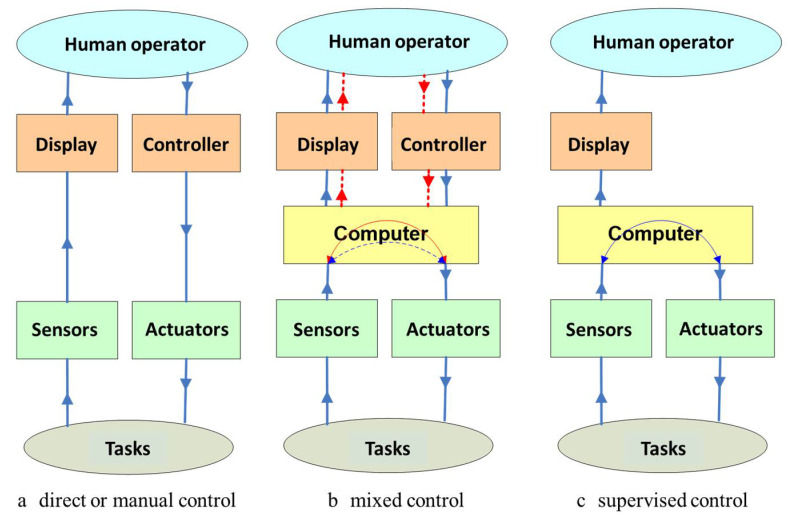
Schematic representation of the three major classes of command-and-control systems of terrestrial robots. For ongoing research, on the use of 6 × 6 terrestrial robots for the purpose of identification, monitoring and intervention in risk areas, we proposed that of the three versions: (**a**) *direct or manual control*; (**b**) *mixed control*; (**c**) *supervised control*, to use version (**b**).

Figure 2 presents the three types of remote-control systems for terrestrial robots, which are currently described in the literature as follows:

- Direct or manual control: The robot does not have artificial intelligence or autonomy, and the operator controls the movement of the robot directly without automatic aid; the principle of operation is very simple and is based on four bits (forward-backward and left-right), in which case signal identification is a priority, as the microcontroller timer is used as an internal counter so that it can calculate the pulse width from the motor shaft [45].
- Mixed control: The robot has a certain degree of artificial intelligence and/or autonomy, and the operator helps it to perform certain operations; to switch to autonomous mode, the system of sensors for orientation (gyroscope) and measurement of distances (odometer) make predictions about the next path to follow. The greater the distance from the operator, the more difficult the teleoperation, especially due to the visual effect on the operator due to the movements of the vehicle and the orientation of the camera. If the orientation of the camera does not correctly compensate for the vehicle’s rotation, it will cause a shunt of the camera and wrong decisions related to the movement of the vehicle [46].
- Supervised control: The robot has a high degree of intelligence and/or autonomy, while the user only intervenes at a high decision level. Control architectures are designed to allow the use of a single control law for each context: the route to the target, the avoidance of obstacles, etc. To manage the interactions between controllers depending on the context, multi-agent systems are used, which can self-organize depending on the emergence of phenomena [47].

**Figure 3:**
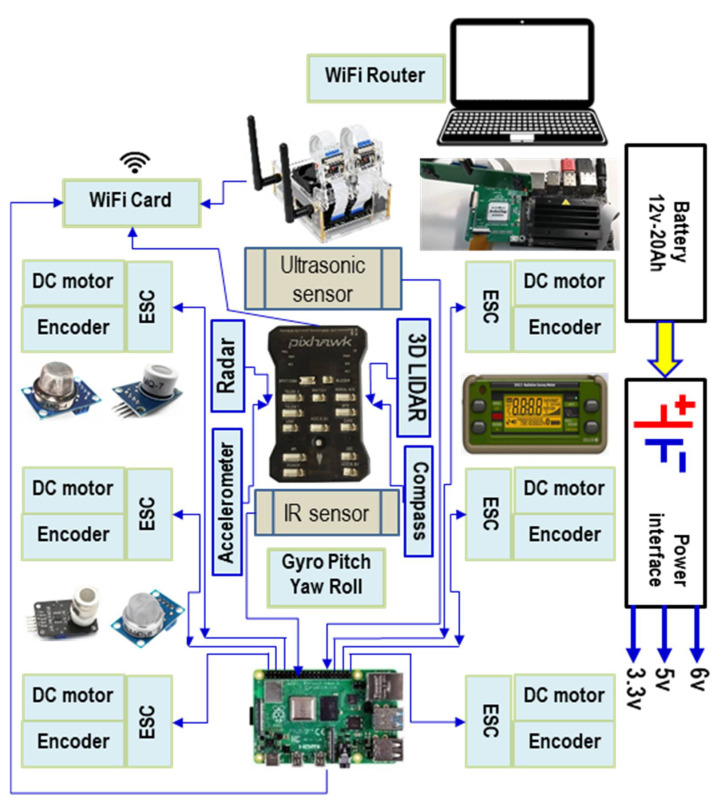
The control and command architecture of the studied robot including obstacle detection (ultrasonic and infrared (IR) sensors); orientation (tri-axial accelerometer and global positioning system (GPS)), gyroscope (pitch, yaw, and roll), compass; DC motor encoder, Electronic Speed Controller (ESC); Pixhawk controller, Raspberry Pi 4 controller and NVIDIA Jetson Nano; power system, and Wi-Fi communication system.

The control architecture for the intervention robot was specific for autonomous navigation and is based on the Raspberry Pi 4, B/4 GB model controllers (for commanding the motors) (Distrelec GmbH Dresdnerstrasse 471200 Wien Austria), NVIDIA^®^ Jetson Nano™ Developer Kit (video image processing) (Distrelec GmbH Dresdnerstrasse 471200 Wien Austria), and Pixhawk Autopilot (navigation) as shown in (Figure 3).

This architecture (Figure 3) was designed to manage the interactions between the elementary controllers. The control laws allow reaching static or moving targets. Regarding the technical detail, the command-and-control system was structured in seven modules:

i. The obstacle detection module, consisting mainly of sensors for measuring distance. The use of several types of sensors (ultrasonic, IR, and 3DLIDAR) was used to allow obtaining values as close as possible to reality. Each type of sensor had its own characteristics regarding the maximum/minimum measuring distance. The 3DLIDAR sensor had the role of performing an overall scan, allowing the lifting of a 3D survey of the area of interest. Overlapping information from the three types of sensors would provide a map of the terrain, including beyond the open view.
ii. The orientation module, composed of inertial sensors, GPS, compass, magnetometer, gyroscope, was used to analyze the position of the robot but also the obstacles to the global and local reference system. This information passed through the displacement algorithm determining the optimal path between the local reference point and the final position. The final position could be variable or fixed. We are talking about fixed targets when we are dealing with stable emergencies. In general, the kinematic and dynamic characteristics of the targets can be variable. For example, the flames of a fire can have different intensities depending on the nature of the combustible material, the direction and speed of the wind, etc.
iii. The motor supply voltage control module consisted of the encoder, and the electronic speed controller (ESC) has the role of modifying the speed and direction of rotation of the motors so that the robot can move on a certain route. It also knows when and how much energy to deliver to the engines when leaving the place or climbing some slopes or climbing some obstacles. In the case of intervention in terrorist actions (artisanal bombs, etc.), the engines must be very stable so that no vibrational phenomena occur. In addition to the traction motors, there will also be motors for the manipulator on the robot.
iv. The module consisted of two types of controllers, Raspberry Pi and Pixhawk, which handled the management of all components of the robot. The Raspberry controller controlled the engines, managed the sensors, the power supply system. The Pixhawk controller handled the management of the navigation system.
v. The module consists of an NVIDIA Jetson nano-controller; two video cameras had the mission of taking video images, analyzing them, and/or sending them wirelessly to the GCS in order to prepare the route to be followed by the robot. The Jeston nano-controller also had a master role.
vi. The power supply module was a structure made up of Li-Ion batteries type 18,650, 5000 mAh, 3.7 V.
vii. Wireless communication module: router and a cheap and available ESP8266 Wi-Fi module with full TCP/IP capability and microcontroller capability. It could be used with any Pixhawk, Raspberry Pi, or Jetson nano-controller.

### 2.3. External Sensor System

The sensor system of the robot consisted of sensors for dangerous substance detection (CO_2_, CO, CH_4_, and H_2_), gamma radiation detection (mass spectrometer), environment conditions determination (meteorological station: humidity, temperature, and air current detection), anemometer, 3D scanning (3D LiDAR), and images (IR/EO video cameras) [48,49,50]. In addition, the intervention for neutralizing an incident with explosive systems involves applying, in all circumstances, some procedures that represent a particular means of action adopted from the specialized personnel in neutralizing/destroying explosive systems or any dangerous material. The intervention robots for missions involving neutralizing explosive devices would be controlled through a remote command or remote control.

Tele-command implies the existence of a barrier between the user and the operating environment in two locations: the operator location and the robot location. The control of the mobile robots, based on remote operation, needs a robust system with an intuitive, flexible, and efficient interface. In the most common situations, mobile robots are equipped with sensors and controllable modules, which provide a significant amount of data for the user [50].

### 2.4. Propulsion System

**Figure 4:**
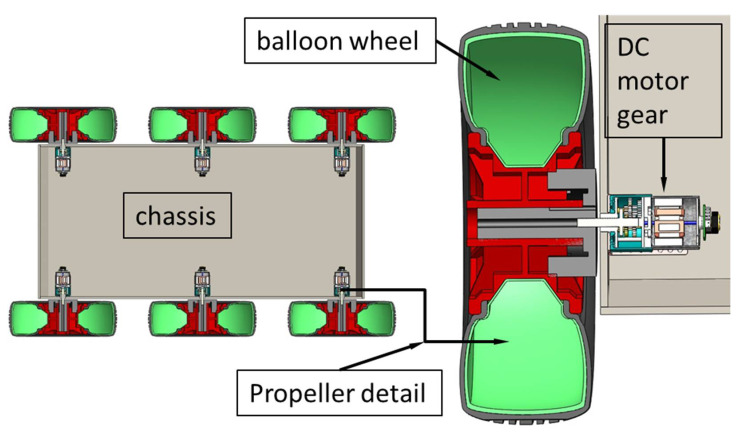
Schematic representation of the propulsion system of the 6 × 6 robot for emergency situations consists of six motors with a gearbox, six wheels with rubber tires, and a nodular balloon inflated with air for more efficient damping of the unevenness of the route during movement of the robot.

The propulsion system consisted of six motors with a JGA25-370 gearbox (6 V, 281 rpm) and six wheels with rims composed of plastic (240 mm diameter, 75 mm tire width, and 20 mm shaft diameter). Each wheel is independent, and the motors were independently driven by electronic stability control (ESC) so that each wheel could be controlled, as shown in Figure 4.

The propulsion solution presented was made in the laboratory. In the future, we propose, together with a potential investor, to replace the wheeled propulsion system with a tracked one, thus reducing the size and gaining space for other components (payload). Replacing the wheeled train with a tracked one would also benefit from the progression in loose terrain or even crossing areas that are full of rubble. The aspect facilitates presented above executing the turns with minimal effort because the propulsion system has only two mobile wheels. The lack of a direction system leads to consistent friction with the ground.

## 3. Analytical Model

The studied robot was a multi-body system (MBS) [51,52] in which the components were categorized as rigid bodies. Researchers [52,53,54] have explained that a multi-body system is a finite set of rigid physically and geometrically interconnected bodies that are also connected to terrain that does not belong to the MBS. The MBS ensemble was built by establishing and imposing some kinematic and dynamic constraints that describe the interconnecting geometrical modes: freedom of degrees, forces, and torque.

To analyze a rigid body in a fixed coordinates system, the mathematical set of the position vectors of the complete structure and/or its components must be determined from a kinematics point of view. Since the environment in which the robot operates is an unstructured environment, the dynamics of the robot should consider the state of terrain, the type of terrain, its density, the loosening of the soil, humidity, etc. [53,54,55].

### 3.1. Kinematics of the Robot with a 6 × 6 Propulsion System

The kinematic model of the robot with six mobile wheels, without any of them being driving wheels, was generated based on some simplifying hypotheses [56,57]. The delay differential equations (DDEs) that describe the initial dimensional structure and use fewer parameters and state variables than the ordinary differential equation (ODE) and partial differential equation (PDE) systems are described in [16].

**Figure 5:**
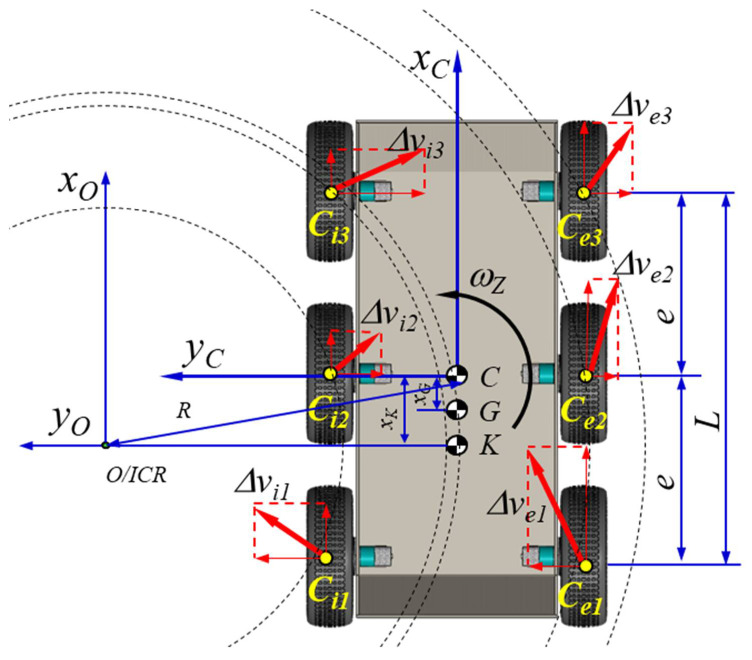
The 6 × 6 kinematic diagrams of the robot showing the following essential characteristics for a simple and fast analysis of the robot kinematics: the six driving wheels are not steering wheels; turns are made by changing the rotational speeds of the six wheels; cornering radii varied depending on the rotational speed of the drive wheels and the indices of adhesion to the ground, the slip indices. If the movement was rectilinear, the decomposition of the speed was uniform only for the ideal conditions of movement; the ground contact of the balloon of the wheels was related to the pressure in the balloon. The weight of the robot and the position of the center of gravity may be altered, in particular, by the operating platforms.

The robot was characterized by non-holonomic behavior because of the effects that appeared at the interaction of the wheels with the rolling path. According to this approach, the equations that describe the direction were differential equations so that the robot had high maneuverability, as shown in Figure 5. In fact, the kinematics diagram was specific to a 6 × 6 vehicle, for which an analytical-experimental model was developed [52,58,59,60]. The viability of the model was verified using a precise laser scanner for localizing the mobile robot in the conditions of the simplified hypotheses [61].

Instantaneous center of rotation (ICR) values are dependent on the dynamics of the robot, which considers the slip ratio, but in an area with medium velocities, once the velocity increases, those values increase too.

For a robot with six mobile wheels, the velocities of the wheels are written as $\left\{\omega_{i}\left|i=1,2,3,4,5,6\right.\right\}$ and the following convention $\left\{\begin{array}{c} \omega_{i/\mathrm{int}}=\omega_{i1}=\omega_{i2}=\omega_{i3}\\ \omega_{e/ext}=\omega_{e1}=\omega_{e2}=\omega_{e3} \end{array}\right\}$. The calculus method of the direct kinematics in the plane can be defined as

(1) $$\left[\begin{array}{c} v_{x}\\ v_{y}\\ \omega_{z} \end{array}\right]=f\cdot \left[\begin{array}{c} \omega_{i}\cdot r\\ \omega_{e}\cdot r \end{array}\right],$$

where v=vx,vy ms is the translational velocity of the robot, ωz rads is the angular rate, and $r\;\left[m\right]$ is the radius of the mobile wheels.

The instantaneous rotation centers are: *ICR*, *ICR_i_*, *ICR_e_*, and *ICR_CG_*; *ICR_i_* and *ICR_CG_* are on a line parallel to the OX axis a line parallel to the OX axis of the Cartesian coordinate system, thus determining the corresponding x and y coordinates:

(2) $$\left\{\begin{array}{l} y_{CG}=\frac{v_{x}}{\omega_{z}}\;\left[m\right]\\ y_{i}=\frac{v_{x}-\omega_{i}\cdot r}{\omega_{z}}\;\left[m\right]\\ y_{e}=\frac{v_{x}-\omega_{e}\cdot r}{\omega_{z}}\;\left[m\right]\\ x_{CG}\equiv x_{i}\equiv x_{e}\equiv -\frac{v_{y}}{\omega_{z}}\;\left[m\right] \end{array}\right.$$

The $J_{\omega }$ matrix is dependent on the ICR coordinates:

(3) $$\left[\begin{array}{c} v_{x}\\ v_{y}\\ \omega_{z} \end{array}\right]\equiv J_{\omega }\equiv \frac{1}{y_{i}-y_{e}}\left[\begin{array}{cc} -y_{e} & y_{i}\\ x_{CG} & -x_{CG}\\ -1 & 1 \end{array}\right]$$

### 3.2. Dynamics of the Robot with a 6 × 6 Propulsion System

**Figure 6:**
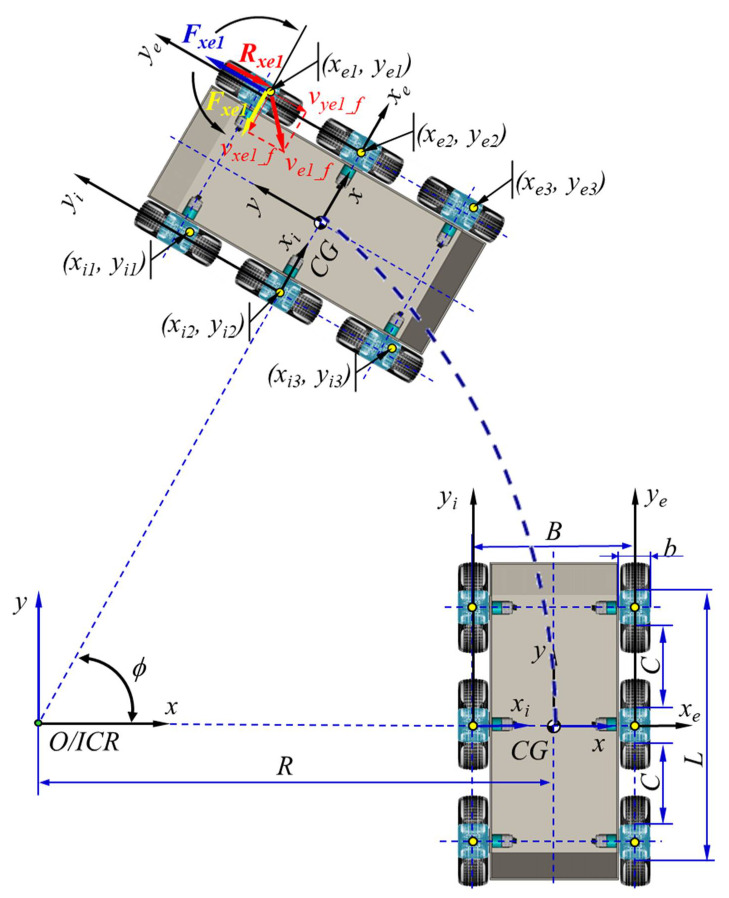
Schematic of the 2D dynamics of the 6 × 6 robot, which had some specificities related to the choice of travel mode. The ground contact spots of the six wheels were identical. Working conditions are ideal when Md = Mr (Md—motor torque; Mr—resistant moment). The geometric center of the contact spots (yellow dots) during the turn is represented outside the geometric center of the contact spots. This representation suggests that progression in the field is influenced by different coefficients and that each operational platform can create an additional balance.

The study of the 6 × 6 dynamics was performed starting from the equations that describe its kinematics [58]. The analytical model shown in Figure 6 describes the movement based on the angular rate of the wheels. This algorithm was chosen because the velocity of the robot is given by the angular rate of the wheels, which were commanded by a controller by varying the input voltage. The dynamics of a robot terrestrial vehicle with six mobile wheels (non-driving) had two calculus models: 2D and 3D. Figure 6 depicts a diagram of the movement on 2D terrain.

#### 3.2.1. D Robotics Dynamics

The dynamic model of the robot is given by the following system of equations relating to the action of the forces upon the propulsion system:

(4) $$\left\{\begin{array}{l} F_{xe1}+F_{xe2}+F_{xe3}+F_{xi1}+F_{xi2}+F_{xi3}-R_{x}-m\cdot \frac{v_{CG}^{2}}{R}\cdot \sin\beta =0\;\left[N\right]\\ F_{ye1}+F_{ye2}+F_{ye3}+F_{yi1}+F_{yi2}+F_{yi3}=m\cdot \frac{v_{CG}^{2}}{R}\cdot \cos\beta \;\left[N\right]\\ M_{d}-M_{r}=0\;\left[\mathrm{Nm}\right] \end{array}\right.$$

where $F_{xe1},F_{xe2},F_{xe3},F_{xi1},F_{xi2},F_{xi3}\;\left[N\right]$ are the longitudinal slipping forces; $F_{ye1},F_{ye2},F_{ye3},F_{yi1},F_{yi2},F_{yi3}\;\left[N\right]$ are the lateral forces; $R_{xe1},R_{xe2},R_{xe3},R_{xi1},R_{xi2},R_{xi3}\;\left[N\right]$ are the drag forces; $M_{d}\;\left[\mathrm{Nm}\right]$ is the motor torque; $M_{r}\;\left[\mathrm{Nm}\right]$ is the resistant moment.

The tension τ (N/m^2^), or the unitary tangential effort in the wheel as a function of the deformation coefficient *j*, is given by

(5) τ=p·μ·1−e−jkγ Nm2,

where p (N/m^2^) is the normal pressure on the terrain, $\mu \;\left[-\right]$ is the friction coefficient, and $k_{\gamma }\;\left[-\right]$ is the coefficient of the shear deformation module.

The longitudinal slip and lateral forces are

(6) $$\left\{\begin{array}{c} F_{xe}=\int_{-\frac{L}{2}}^{\frac{L}{2}}\int_{-\frac{b}{2}}^{\frac{b}{2}}p_{e}\cdot \mu_{e}\cdot \left(1-e^{-\frac{j_{fe}}{k_{\gamma e}}}\right)\cdot \sin\left(\pi +\omega_{\gamma e}\right)\cdot dx_{e}\cdot dy_{e}\;\left[N\right]\\ F_{xi}=\int_{-\frac{L}{2}}^{\frac{L}{2}}\int_{-\frac{b}{2}}^{\frac{b}{2}}p_{i}\cdot \mu_{i}\cdot \left(1-e^{-\frac{j_{fi}}{k_{\gamma i}}}\right)\cdot \cos\left(\pi +\omega_{\gamma i}\right)\cdot dx_{i}\cdot dy_{i}\;\left[N\right] \end{array}\right.$$

(7) $$\left\{\begin{array}{c} F_{ye}=\int_{-\frac{C}{2}}^{\frac{C}{2}}\int_{-\frac{b}{2}}^{\frac{b}{2}}p_{e}\cdot \mu_{e}\cdot \left(1-e^{-\frac{j_{fe}}{k_{\gamma e}}}\right)\cdot \sin\left(\pi +\omega_{\gamma e}\right)\cdot dx_{e}\cdot dy_{e}\;\left[N\right]\\ F_{yi}=\int_{-\frac{C}{2}}^{\frac{C}{2}}\int_{-\frac{b}{2}}^{\frac{b}{2}}p_{i}\cdot \mu_{i}\cdot \left(1-e^{-\frac{j_{fi}}{k_{\gamma i}}}\right)\cdot \cos\left(\pi +\omega_{\gamma i}\right)\cdot dx_{i}\cdot dy_{i}\;\left[N\right] \end{array}\right.$$

where $j_{fe},j_{fi}\;\left[-\right]$ is the movement of each wheel and ωγe,ωγi rads is the angular velocity of the wheels.

#### 3.2.2. D Robotics Dynamics

The dynamics of a terrestrial vehicle in an unstructured environment has a non-linear and varying characteristic in time. When the robot moves on tracks with slope α, its total weight $G_{a}\;\left[N\right]$ can be separated into a component that is parallel to the rolling path $R_{p}=G_{a}\cdot \sin\alpha \;\left[N\right]$ (when it climbs a slope, its sign is negative as it opposes the movement of the robot; when it descends a slope, its sign is positive because the component becomes an active force) and a component perpendicular to the rolling path $R_{r}=G_{a}\cdot \cos\alpha \;\left[N\right]$.

**Figure 7:**
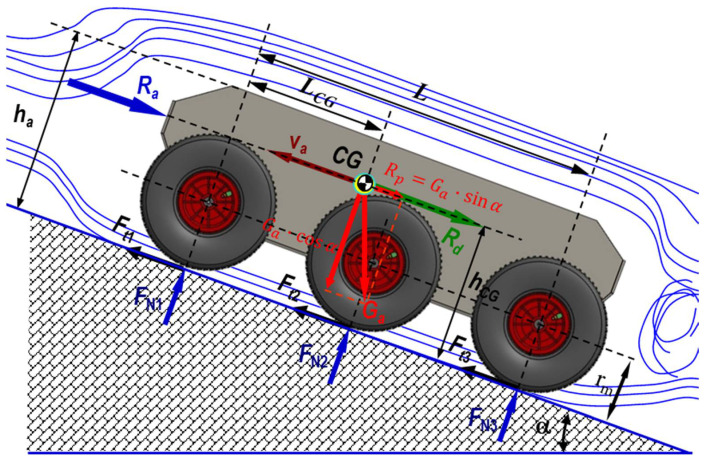
A schematic of the 3D dynamics of the 6 × 6 robot showing the elements that influence the dynamic characteristics of a land vehicle: air resistance, friction force, robot weight, center of gravity position, wheel radius, and slope angle. The following figures will highlight other elements that contribute to influencing the law of motion of the robot.

Figure 7 depicts the schematics of the drag at climbing the slope $R_{\psi }\;\left[N\right]$

(8) $$\left\{\begin{array}{l} R_{p}=\pm G_{a}\cdot \sin\alpha \\ R_{r}=f\cdot G_{a}\cdot \cos\alpha \end{array}\right.\;\Rightarrow \;R_{\psi }\equiv R_{p}+R_{r}\equiv G_{a}\cdot \left(\pm \sin\alpha +\cos\alpha \right)\;\left[N\right]$$

(9) $$R_{d}=\delta \cdot \frac{G_{a}}{g}\cdot \frac{dv}{dt}\;\left[N\right]$$

where $R_{d}\;\left[N\right]$ is the start drag, $\delta \;\left[-\right]$ is the coefficient of influence of the masses in rotation, and $f\;\left[-\right]$ refers to the total coefficient of friction

(10) $$R_{a}=K\cdot A\cdot v_{a}^{2}$$

where $R_{a}\;\left[N\right]$ is air drag, K=0.6125·cx kgm3 is the aerodynamic coefficient in standard aerodynamic conditions, $c_{x}\;\left[-\right]$ is the air drag coefficient, and $A\;\left[m^{2}\right]$ is the transversal surface of the robot.

**Figure 8:**
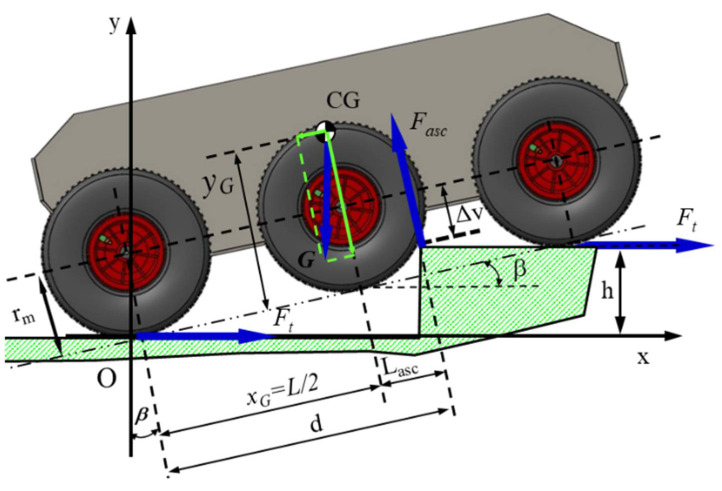
Schematic of the escalation of a step-type obstacle by the robot. To ensure good ability to climb the step, the front, and middle wheels to be in permanent contact with the step to be climbed. It is not mandatory to have this situation; depending on the geometry of the workspace, energy consumption can increase, which affects the autonomy of movement. The robot was designed to move without being powered by a cable.

Taking this perspective, Figure 8 depicts the schematics of climbing a step obstacle. Applying the Equations (11)–(14) regarding scaling an obstacle in steps, we obtained the friction coefficient corresponding to the step obstacle

(11) $$\mu \cdot d=r_{m}\cdot \sin\beta +x_{G}\cdot \cos\beta -y_{G}\cdot \sin\beta \;\left[m\right]$$

(12) $$\mu \cdot h=r_{m}-r_{m}\cdot \cos\beta +d\cdot \sin\beta \;\left[m\right]$$

(13) $$d=\frac{\left(h-r_{m}+r_{m}\cdot \cos\beta \right)}{\sin\beta }\;\left[m\right]$$

(14) $$\mu =r_{m}\cdot \sin\beta +d\cdot \cos\beta \;\left[m\right]$$

where $x_{G}\;\left[m\right]$ is the distance from the rear wheel shaft to the center of mass, measured on the direction of the imaginary line that passes through the center of the wheels; $y_{G}\;\left[m\right]$ is the distance from the center of mass to the imaginary line that passes through the center of the wheels; $r_{m}\;\left[m\right]$ is the radius of the wheels; $h\;\left[m\right]$ is the height of the step obstacle; $d\;\left[m\right]$ is the length of the imaginary line between the rear contact point and the edge of the obstacle (m); is the tilt angle of the shaft of the robot; $\mu \;\left[-\right]$ is the friction coefficient corresponding to the step obstacle.

**Figure 9:**
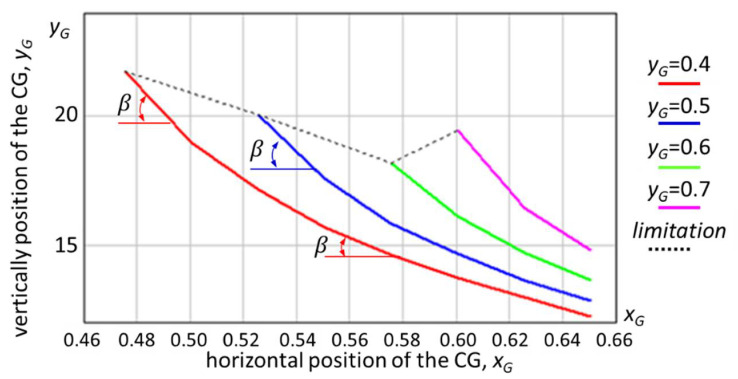
The variation in $\beta \;\left[grd\right]$ as a function of the center of mass for h = 0.20 m (where h is the height of the step obstacle); the height of the step is constant. If the step to be scaled has a different height, the values in the graph will change. The pressure value of the wheel tires was not considered in this simulation.

Following the application of Equations (11)–(14), it was possible to obtain the curves of variation in the angle of inclination of the towing platform through an analytical-numerical simulation, depending on the position of the center of gravity for the height (considered constant) of the step to be scaled, which is highlighted in Figure 9.

Increasing the height of the ground robot’s center of gravity would allow it to tackle higher obstacles. However, obviously, for the accuracy of the calculation algorithm, the fact that the angle of approach of an obstacle increases means that the size of the drive wheel becomes important, and the distance between the axle of the front axle and the rear axle is also important. For example, there may be situations where a large wheelbase does not allow cornering, so the robot would not be prevented from completing the mission.

## 4. Simulation of the 6 × 6 Propulsion System

### 4.1. Simulation of Slope Climbing

**Figure 10:**
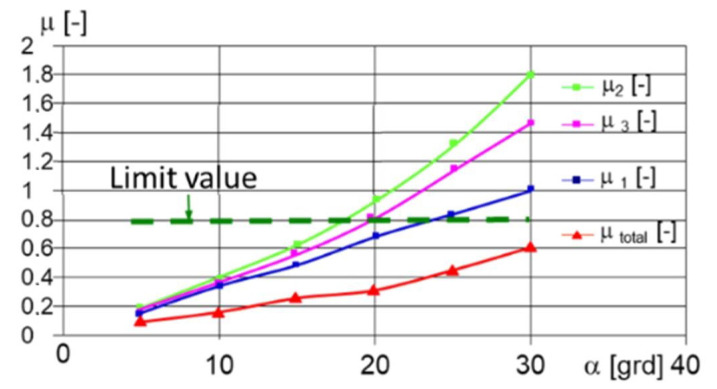
The variation in the $\mu \;\left[-\right]$ necessary for climbing a slope following the simulation using Equations (15)–(19). For the geometric characteristics of the robot and the total minimum adhesion coefficient, the robot could climb a step under the conditions of a CG (center of gravity of the robot) with a height = 0.20 m.

**Figure 11:**
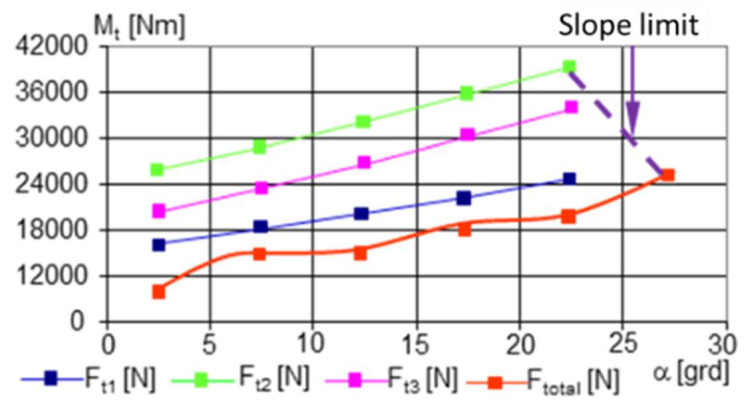
The variation in the necessary $M_{t}\;\left[Nm\right]$ as a function of the tilt angle of the slope.

To evaluate the potential to simulate the slope climbing, for longitudinal movement on a slope, the following must be considered: $m\;\left[kg\right]$, the mass of the robot; $F_{t}\;\left[N\right]$, the thrust force; $F_{N}\;\left[N\right]$, the normal force; $\alpha \;\left[grd\right]$, the tilt angle of the longitudinal slope;$L_{CG}\;\left[m\right]$, the distance between the center of mass and the shaft of the front deck; *h_cg_*, the distance between the center of mass and the rolling path surface (m); $L\;\left[m\right]$, the distance between the shafts; $\mu \;\left[-\right]$, the friction coefficient corresponding to the tilt angle of the slope (Figure 10); $M_{t}\;\left[Nm\right]$ the moment for climbing the slope (Figure 11).

The equations for the normal forces that act upon the wheels and for the minimum adhesion coefficient, which ensures using the thrust forces, are given in Equations (15)–(19):

(15) $$F_{N}=\frac{m\cdot g\cdot \left(L_{CG}\cdot \cos\alpha +h_{CG}\cdot \sin\alpha \right)}{L}\;\left[N\right]$$

(16) $$F_{t}=m\cdot g\cdot r_{m}\cdot \frac{\left(\sin\alpha +f\cdot \cos\alpha \right)}{6}\;\left[N\right]$$

(17) $$M_{t}=m\cdot g\cdot r_{m}\cdot v_{a}\cdot \frac{\left(\sin\alpha +f\cdot \cos\alpha \right)}{6}\;\left[Nm\right]$$

(18) $$\mu \geq \frac{L\cdot \sin\alpha }{h_{CG}\cdot \sin\alpha +L_{CG}\cdot \cos\alpha +L\cdot \cos\alpha }\;\left[-\right]$$

(19) $$\alpha =\arctan\left(\frac{L-L_{CG}}{h_{CG}}\right)\;\left[rad\right]$$

The graphs in Figure 10 describe how the minimum coefficient of adhesion varies depending on the angle of inclination of the longitudinal slope. It can be easily observed that with the multiplication of the number of drive axles (two drive wheels for one axle), the grip capacity improved.

For the same geometric conditions and coefficients of friction with the ground (respectively, with the climb), Figure 11 displays the variation in the moment for climbing the slope.

Similar to Figure 10, the graph in Figure 11 shows how the effort is exerted by the engine, once it is distributed on several drive wheels, which has the positive effect of reducing energy consumption, thus increasing the power reserve for various unforeseen situations.

### 4.2. Simulation of Crossing a Step-Type Obstacle

**Figure 12:**
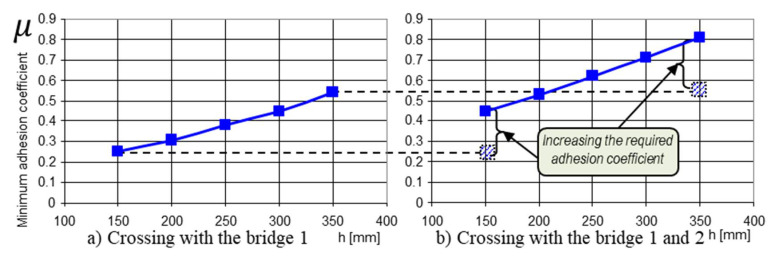
The variation in the $\mu \;\left[-\right]$ necessary to cross an obstacle as a function of its height. The data used for the calculations are found in Appendix A and Table A1. Given the geometric characteristics of the towing robot and the mobile platform, and that changes are possible depending on the dimensions of the operational platforms, the robot can be used for different missions; we consider the data used to be sufficient: (**a**) crossing with bridge 1; (**b**) crossing with bridges 1 and 2.

**Figure 13:**
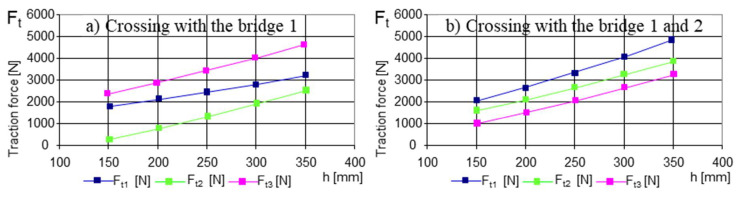
The variation in the $F_{t}\;\left[N\right]$ necessary for crossing an obstacle as a function of its height. The explanations presented in Figure 12 also apply here: (**a**) crossing with bridge 1; (**b**) crossing with bridges 1 and 2.

Crossing a step-type obstacle was simulated in two variants (Appendix A and Table A1):

- The obstacle was approached with both front deck wheels (Figure 12)
- The obstacle was approached with four wheels corresponding to the 1 and 2 decks (Figure 13)

Although, apparently, the index of the value of the minimum coefficient of adhesion provides information on the ability of a land vehicle to move, i.e., to be able to cling to the ground for progression, the graphs in Figure 12 provides precious information about when the robot has to climb an obstacle. In other words, the difference between the graphs in Figure 12a,b is obvious; with a safe drive axle (two-wheel drive), the climbing capacity was much lower, and energy consumption was increased.

With the escalation of an obstacle, the driving wheels would be in contact with it, successively (from the point of view of the obvious cinematic). But we could observe the distribution of the traction force on each drive axle (two conjugate drive wheels) Figure 13a. When the motor and the second axle came into contact with the obstacle, there was a new distribution of the traction forces, see Figure 13b. The new values of the traction forces showed that with the unloading of the weight force on another drive axle, the third axle required less pushing.

## 5. Testing the Braking When Descending a Slope in Dynamic Mode

**Figure 14:**
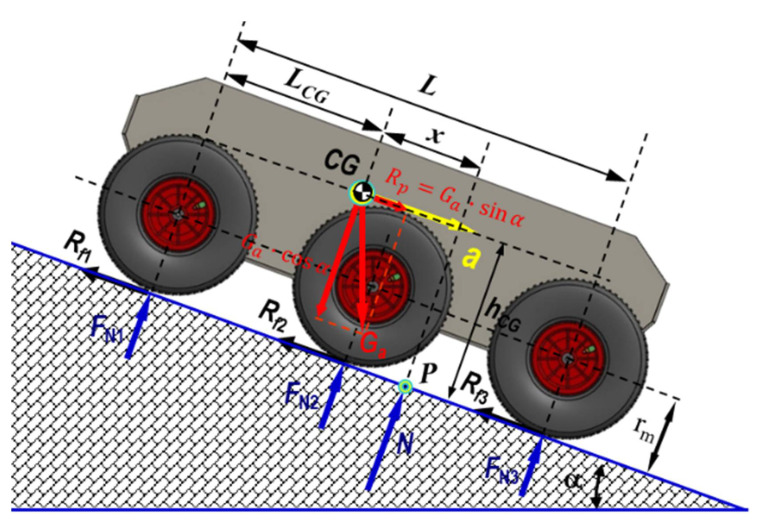
Schematic of braking while descending a longitudinal slope. The dynamics of the robot during braking are much more suggestive when the process is diagrammed during the descent of a slope. In this case, two other notions intervene: *a,* deceleration when braking; $R_{f}$, friction force.

The geometrical characteristics of the propulsion system, as the position of the center of mass of the mobile robot, are critical for ensuring stability in a static regime (Figure 14).

From the viewpoint of the stability of the platform, while moving on a longitudinal slope, as outlined in Figure 14, the most difficult situation occurred during braking while descending on a slope, where $a=\frac{d^{2}x}{dt^{2}}\;\left[m/s^{2}\right]$ represents the deceleration and $R_{f}\;\left[N\right]$ is the braking force.

**Figure 15:**
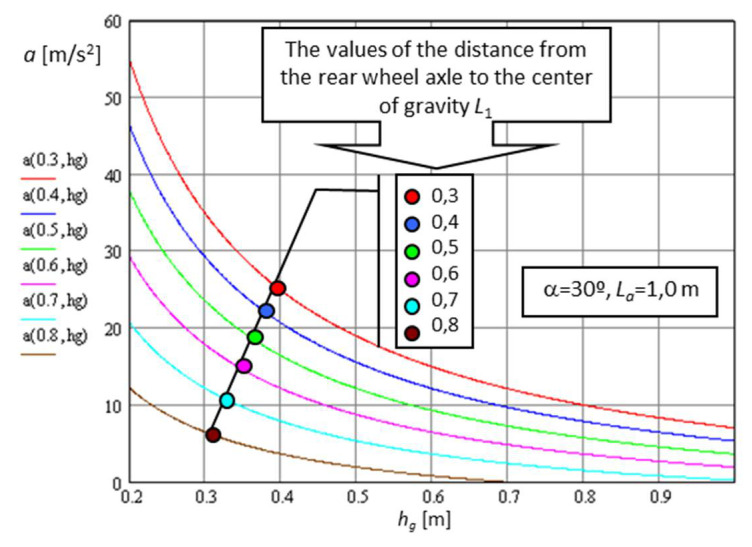
The variation in the maximum deceleration at braking on a 30º slope as a function of the center of mass.

As can be seen in Figure 15, At the limit, the x misalignment of the pressure center on the terrain becomes equal to $x=L-L_{CG}$, where, after solving the equilibrium equation of the torques around the P point, the deceleration becomes:

(20) a=d2xdt2=g·L−LCGhCG·cosα−sinα ms2

Thus, both increasing $h_{CG}$ and moving it toward the front of the platform leads to decreasing the deceleration at braking because rollover could occur.

Robots with all the mobile wheels driven by electrical motors can brake by stopping the power supply or blocking the area of contact of the tire with the terrain; thus, at the limit, Equation (20) becomes:

(21) a=d2xdt2=g·L−LCGhCG ms2

After observing the measurements acquired by the accelerometers, we estimated that the analytical-numerical model would allow us to use it for the software necessary for autonomous navigation.

**Figure 16:**
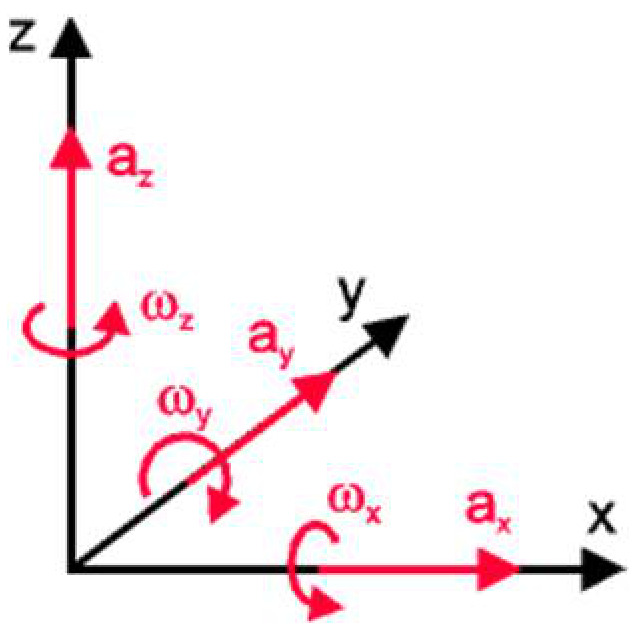
Basic reference system for inertial navigation.

For clarity, the position of the body at a given time can be determined by integrating the accelerations measured in three mutually perpendicular directions and the angular velocities of rotation around the same axes (Figure 16).

**Figure 17:**
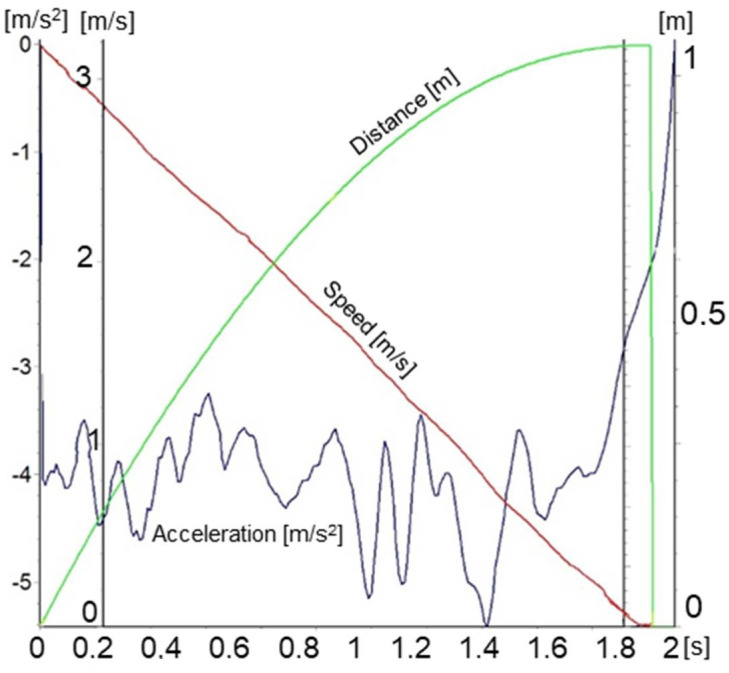
The dynamic characteristics of the 6 × 6 robot when braking on the slope, determined with the help of an inertial sensor system.

In Figure 17, the dynamic characteristics of the 6 × 6 robot when braking on the slope are determined with the help of an inertial sensor system.

The absolute speed measurement system based on inertial navigation was adopted because it allows the determination of the absolute speed of the robot. By integrating the data on accelerations and angular velocities, we determined the trajectory of the center of gravity.

**Figure 18:**
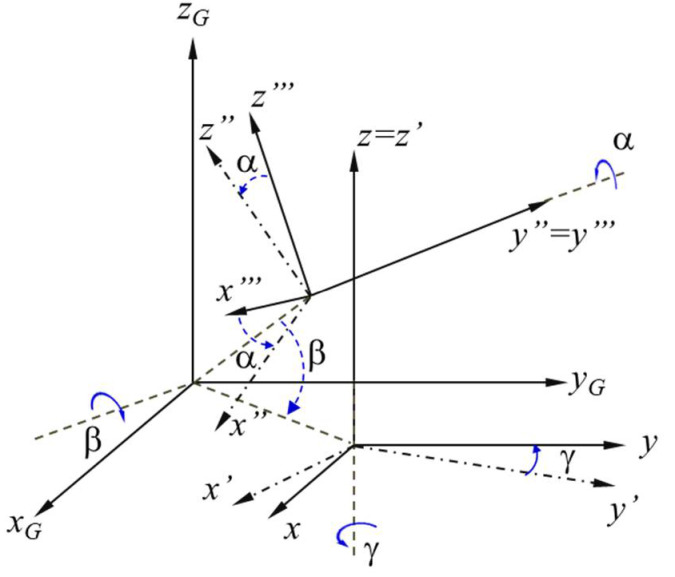
Representation of the displacement of the coordinate system with the help of Euler’s angles.

The main limitation of this type of absolute speed measurement is the sensitivity and accuracy of the sensors used to measure accelerations and angular velocities. As high-precision sensors are expensive and require sophisticated calibration programs, a cheap calibration solution for the digital inertial sensor was adopted. We also considered the average of the vertical acceleration (along the z-axis) as equal to the gravitational acceleration. A GPS sensor was also used to increase the accuracy of the results. For this, the calibration solution consisted of applying the corrections for moving in three-dimensional space using the representation based on Euler’s angles and Euler’s rotation theorem, as can be seen in Figure 18.

**Figure 19:**
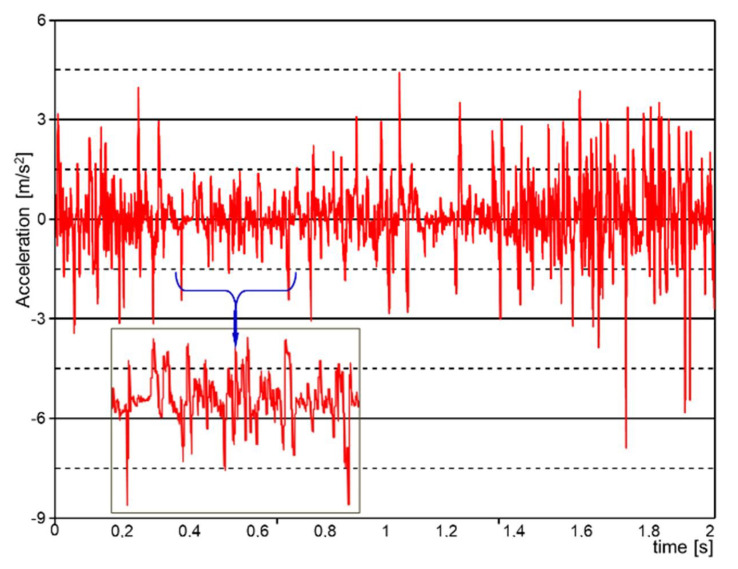
The evolution of the longitudinal acceleration while braking on a slope, determined with the help of a GPS sensor system.

In Figure 19, the longitudinal acceleration was determined using the derivative of the velocity, followed by filtering the resulted signal.

The importance of determining or measuring the acceleration during the braking of a vehicle is given primarily by the fact that we can determine the braking space. On the other hand, if the robot’s operating platform is equipped with, for example, a manipulator arm, the deceleration value helps us to determine how long the elements of the arm can be and what payload it can develop under certain travel conditions. Also, when using the robot to move dangerous substances or even an artisanal bomb equipped with vibration sensors, we must know the instantaneous variations of deceleration and acceleration.

## 6. Discussion of Potential Emergency Response Missions

The platform/carrier vector is the robot chassis, which was equipped with the propulsion, the command-and-control, and power supply systems. The platform/operational vector is a structure that can be interchangeable. Depending on the missions to be performed, the carrier vector can receive an operational vector. Next, we present two of the equipment possibilities for emergency interventions that we studied.

### 6.1. Emergency Response to Fires

**Figure 20:**
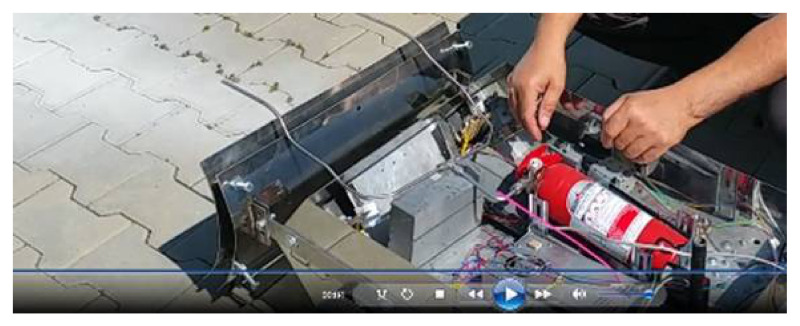
Fire extinguishing system with two fire extinguishers and mechanism for maintaining the pressure of the extinguishing-type powder (Florex, E.12, Carbo, based on KHCO_3_).

The emergency response to fires requires an operational vector that can perform measurements on environmental conditions, the temperature of the affected area, and the types of harmful substances and their concentrations. Figure 20 depicts the elements that comprised a fire extinguishing system, which was moved to the risk area using a wheeled robot.

**Figure 21:**
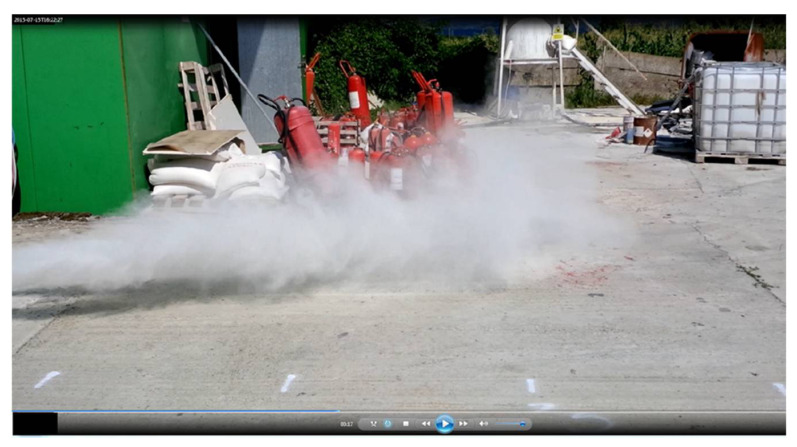
Extinguishing jet performance. The fire was maintained by a pile of cotton cloths soaked in liquid fuel or diesel. When the smoke became sufficient and the wind direction changed toward the robot, the smoke sensors triggered the fire extinguishing system. The system was constructed with the help of two inert gas fire extinguishers.

After testing the fire extinguishing system, using a fire set with a cloth soaked in diesel, we examined the capacity of the robot to intervene in this scenario, so we list in Figure 21.

**Figure 22:**
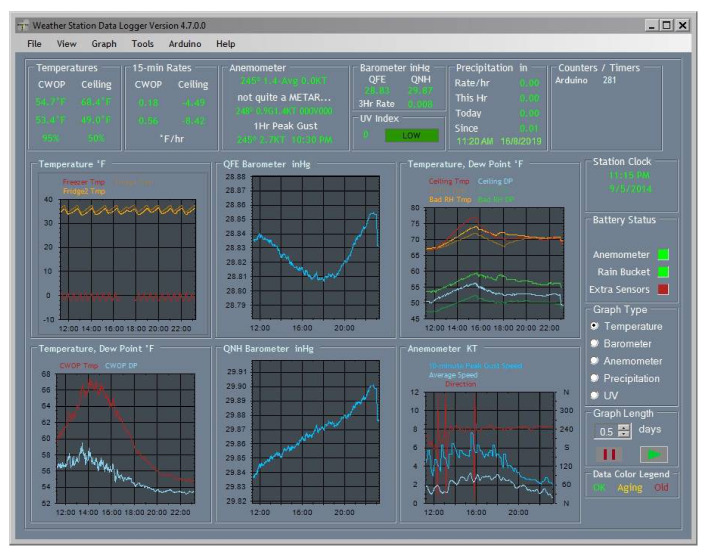
Environmental samples taken with an Arduino-controlled weather station *p*/*n* 80,422. The values recorded by the weather station correspond to a summer period.

Another aspect regarding the safety of the robot and the equipment of the robotic system is a flame protection shield, which was equipped to allow the robot to approach as close as possible to the fire. To highlight the characteristics of the elements that can influence the distribution and intensity of a fire, Figure 22 shows the data recorded with an Arduino-controlled weather station.

**Figure 23:**
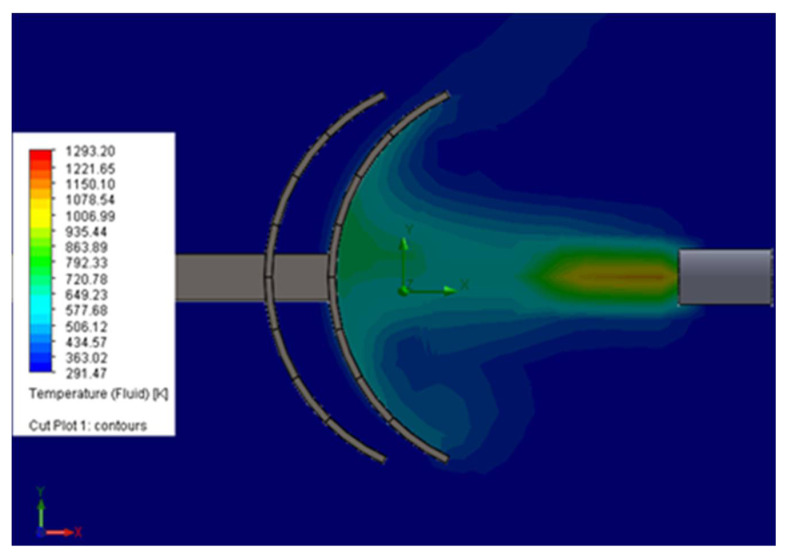
Air temperature simulation for an output speed of 10 m/s. The numerical simulation was performed using the finite element simulation application Solid Works.

Figure 23 shows the simulation of the effects of high flame temperatures on the shield. As the purpose of this part of the study was to examine the effects of fire on the robot, we briefly present the concerns regarding the interventions that the robot could perform in emergency situations.

**Figure 24:**
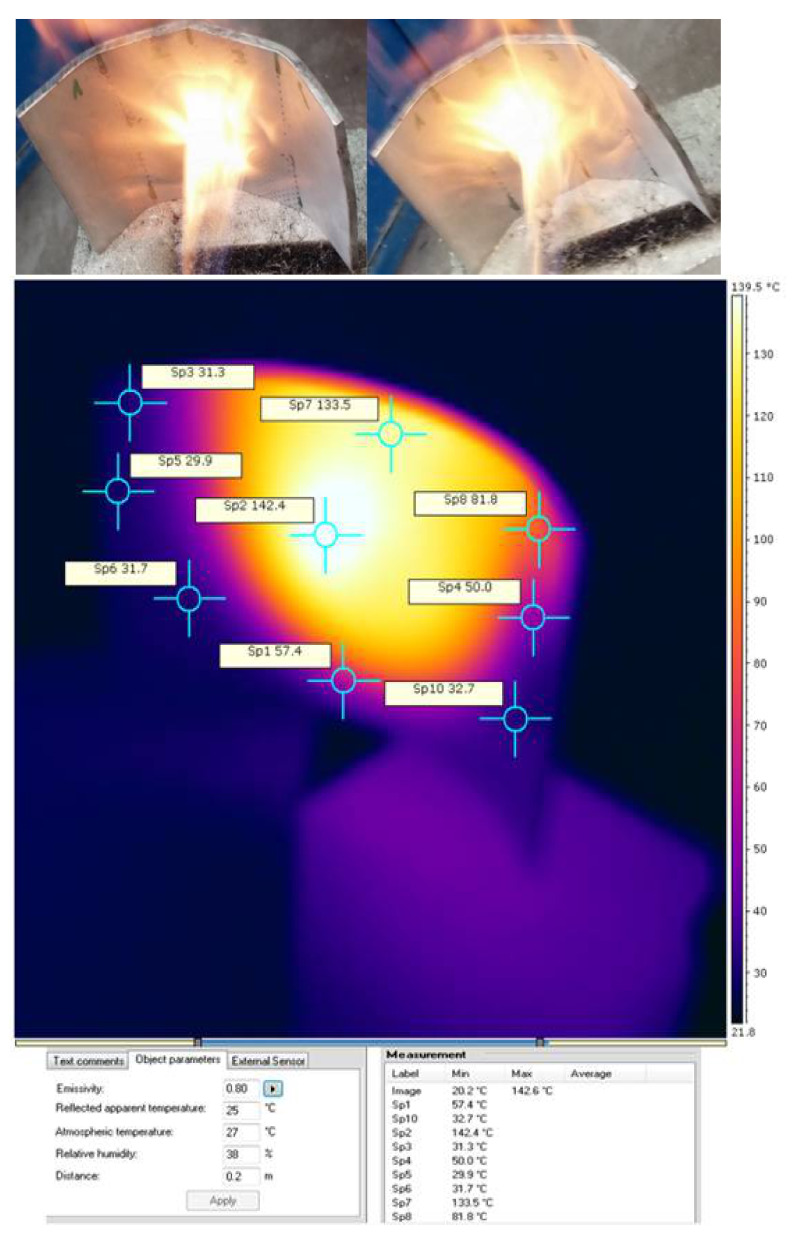
Thermal study for target C (front view) direct contact with the flame at a temperature above 150 °C. Plate thickness: 4 mm, material: 316 L stainless steel, holding time: 4 min, and a concave shape.

After testing the protection system with a stainless-steel shield, as shown in Figure 24, we verified the values obtained by numerical simulation.

### 6.2. Intervention in Emergencies Due to Gamma Radiation Emissions

An important issue during this experimental study was the use of modern solutions to ensure the transport of the measuring system through remote control systems such as robots. Some of the most important advantages of using a robotic system during a measurement operation in high-risk environments are the following:

- Improving safety by eliminating direct exposure of personnel to possible hazardous radiation.
- Reduction in operating time.
- The accuracy and precision of providing information.
- Reducing costs to help minimize the social and economic impact of possible accidents.

**Figure 25:**
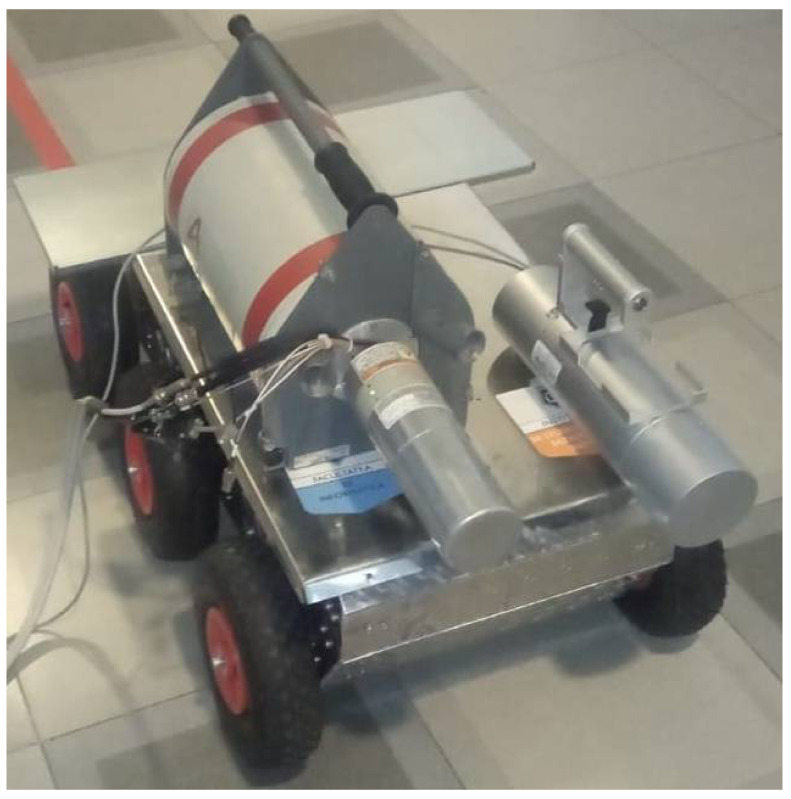
Robotic system with an operational vector used to determine gamma radiation. The experiments were recorded at Titu Maiorescu University in Bucharest.

To minimize the risk of human exposure in hazardous environments, we aimed to perform an experiment consisting of transporting the detection equipment with a remote-controlled robot. Thus, the robot carried the equipment that was the subject of the first experiment on rough terrain to capture specific measurements to determine the radioactivity of the environment (Figure 25).

**Figure 26:**
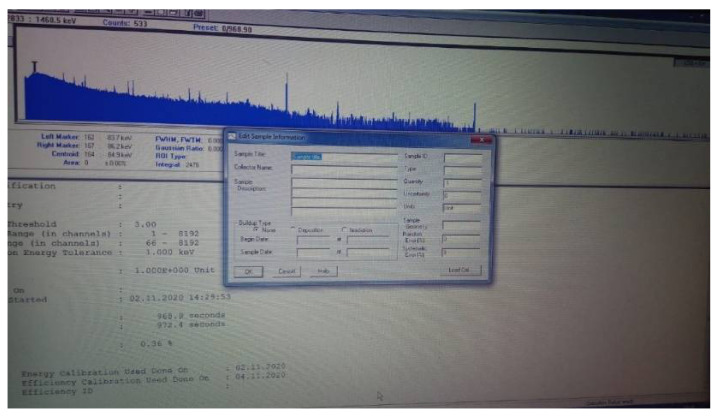
Gamma radiation measurement with a Canberra dosimeter and spectrometer. The recorded values were part of the background of natural radiation. The values that stood out were specific to potassium. The purpose of data collection was to demonstrate the robot’s ability to perform different missions.

The data from Figure 26 are transmitted remotely via a secure connection from a laptop connected to the multichannel analyzer (MCA) and retrieved by specialized personnel to process it and make the necessary decisions.

The representation in Figure 26 is a photo of data from the Canberra system, highlighting what can be measured with it.

## 7. Conclusions

Consistent with the results obtained, we found that our choice of geometric dimensions was appropriate as the values regarding the robot dynamics (Figure 10, Figure 11, Figure 12 and Figure 13), which were determined analytically, satisfy the mobility and stability requirements.

As the height of the center of mass increased, the characteristics when climbing stepping-type obstacles improved, but this advantage, above certain values (Figure 15), generated a loss of stability if the robot moved on a slope. This worsened under braking conditions when descending a slope (Figure 17).

Variations in acceleration and deceleration (Figure 17) lead to corresponding variations in traction forces and traction moments, which were identical to those that reflect the variation in acceleration. The variation in the traction forces, especially when turning, was influenced by the length of the adhesion surface because, with the increase in the width of the point of contact of the tire with the ground, the average pressure on the ground decreased, and the engine simultaneously sank, especially if the ground was deformable. Reducing the average pressure on the ground, even if it reduced the diving effect during a turn, also led to a reduction in the effect of lateral excavation. Another effect of reducing diving when the pressure on the ground decreased was that the grip decreased, so the wheel no longer gripped the ground very well. This side effect did not result in a significant reduction in traction (Figure 11). Overall (Figure 13), the total traction force was sufficient to move on soft ground and climb obstacles.

Inertial acceleration measurement systems are suitable for measuring decelerations/accelerations regardless of the type of rolling. The solution implemented with inertial sensors proves its effectiveness, especially when the robot is moving on rough terrain (specific to an unstructured environment). These results also reaffirm the idea that if the missions could be conducted in screened areas where satellite systems have no signal (caves, salt mines, subway, etc.), the inertial sensory system would allow the identification of specific parameters.

The processing of data captured by inertial sensors and using a GPS positioning system would be able to accurately determine the lateral sliding angle. The statement is based on the positioning error caused by the GCS (fixed station) being transmitted wirelessly to the robot so that the controller will be able to make the necessary corrections.

## Data Availability

Not applicable.

## Appendix A

**Table A1:** The characteristics of the robot used for the simulation.

Radius of the wheels $r_{m}\;\left[mm\right]$	130.00
Wheelbase $L\;\left[mm\right]$	400.00
Distance between the front deck and the center of mass $L_{CG}\;\left[mm\right]$	200.00
Height of the center of mass related to the rolling path $h_{CG}\;\left[mm\right]$	125.00
Mass of the unequipped robot $m\;\left[kg\right]$	35.00
Adhesion coefficient $\mu \;\left[-\right]$	0.8
